# *PRNP* E146G mutation inherited prion disease: distinctive clinical, pathological and fluid biomarker features

**DOI:** 10.1007/s00415-025-13022-2

**Published:** 2025-03-29

**Authors:** Thomas Coysh, Zane Jaunmuktane, Laszlo L. P. Hosszu, Nour Majbour, Fuquan Zhang, Tracy Campbell, Lee Darwent, Marcelo Barria Matus, Edgar Chan, Leah Holm-Mercer, Tze How Mok, Jonathan D. F. Wadsworth, Jan Bieschke, Kannan Nithi, Sebastian Brandner, Colin Smith, Margaret Esiri, John Collinge, Simon Mead

**Affiliations:** 1 MRC Prion Unit at UCL, UCL Institute of Prion Diseases, 33 Cleveland Street, London, W1W 7FF UK; 2 National Prion Clinic, University College London Hospitals NHS Foundation Trust, National Hospital for Neurology and Neurosurgery, Queen Square, London, WC1N 3BG UK; 3 Department of Neuropathology, National Hospital for Neurology and Neurosurgery, Queen Square, London, WC1N 3BG UK; 4 National CJD Research & Surveillance Unit, Centre for Clinical Brain Sciences, University of Edinburgh, Edinburgh, EH16 4SB UK; 5 Department of Neurology, Northampton General Hospital NHS Trust, Cliftonville, Northampton, NN1 5BD UK; 6 Department of Neuropathology, West Wing, John Radcliffe Hospital, Headley Way, Headington, Oxford, OX3 9DU UK

**Keywords:** Inherited prion disease, Prion, Neurodegeneration, Biomarkers, Pathology, Mutation

## Abstract

Inherited prion diseases (IPDs) are phenotypically diverse neurodegenerative conditions caused by mutations in the prion protein gene (*PRNP*). We describe IPD due to a novel *PRNP* E146G mutation in a 50-year-old man presenting with slowly progressive dysarthria, prominent myoclonus especially in the lower limbs, and less prominent gait ataxia, pyramidal and extrapyramidal signs. Cognitive impairment was not overt at disease onset. MRI revealed cerebellar atrophy and white matter hyperintensities. His 46-year-old sister carries the mutation and has subtle gait ataxia and dysarthria. Both patients exhibit a distinctive fluid biomarker profile: in CSF S100B is > twofold upper limit of normal, total tau is moderately elevated, and neurofilament light chain, 14-3-3 and RT-QuIC are negative; in plasma there is marked elevation of GFAP but repeatedly normal neurofilament light chain. The proband’s father died aged 55 following an 8-year dementing illness with similar presentation. Post-mortem revealed cerebellar cortical atrophy and profuse large PrP amyloid plaques across cerebral and cerebellar grey matter. Immunoblotting identified low molecular weight protease-resistant PrP fragments. E146G mutation IPD broadly fits into the historical Gerstmann–Sträussler–Scheinker disease spectrum but, based on deep clinical phenotyping of this initial pedigree, we highlight some distinctive features, which may aid in identification of this disease.

## Introduction

Prion diseases are universally fatal and transmissible neurodegenerative diseases, with a common mechanism of seeded polymerisation of prion protein (PrP) into protease-resistant disease-associated forms. Inherited prion diseases (IPDs) are a phenotypically diverse group of neurodegenerative diseases caused by mutations in the prion protein gene, *PRNP*, which are not always straightforward to identify clinically and can mimic other gradually progressive neurodegenerative diseases [21]. Prion diseases account for approximately 1 in 5000 deaths in the UK, of which IPDs constitute 10–15% [27]. Timely diagnosis of IPDs is of increasing importance as we move towards clinical trials of rationally designed treatments for prion disease [5, 23].

Historically, IPDs have been divided into 3 groups based on clinical syndromes: familial Creutzfeldt–Jakob disease (CJD) associated with rapidly progressive neurocognitive decline and generally readily identified clinically in combination with modern CSF and MRI investigations; fatal familial insomnia (FFI) due to D178N-129M associated with insomnia, hallucinations, dysautonomia and motor signs and Gerstmann–Sträussler–Scheinker disease (GSS), originally reported in the context of P102L mutation, associated with gradually progressive lower limb sensory impairment and an ataxic illness, followed later by cognitive decline [8, 21]. However, this historical classification system does not adequately represent the breadth of distinct clinicopathological phenotypes now appreciated, including IPD due to octapeptide repeat insertions in the prion protein, typically presenting with a gradually progressive dementia with executive dysfunction and dyspraxia [6, 21], *PRNP* point mutations such as P105L and T107I mimicking early-onset Alzheimer’s disease [13, 16] and PrP systemic amyloidosis due to C-terminal *PRNP* truncation mutations, typically presenting with chronic diarrhoea, length-dependent sensory and autonomic neuropathy followed later by gradual cognitive decline [22]. This report adds further to the breadth of known IPDs and highlights the importance of *PRNP* sequencing in unexplained neurodegenerative disease.

## Methods

### Patients

**Fig. 1:**
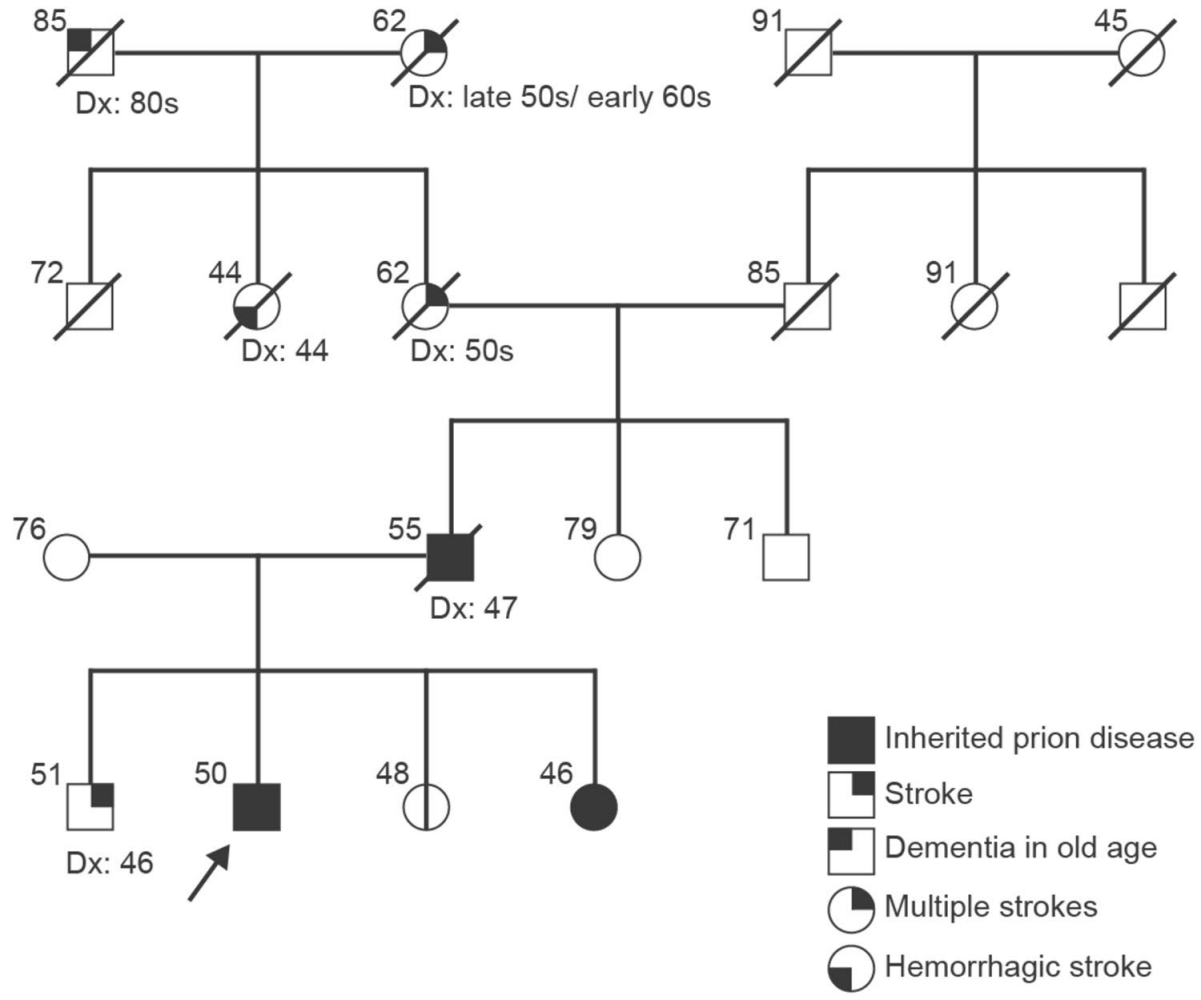
Pedigree including the E146G proband (indicated with an arrow) noting any neurological disease, indicated by different symbols in the legend, current age (or age at death) above and age at disease onset (Dx) below

The proband and his sisters were assessed as part of the National Prion Monitoring Cohort research study (Scotland A Research Ethics Committee (Ref: 05/MRE/0063)). The proband’s father’s clinical information was gathered through interview with family members and case note review. The pedigree is displayed in Fig. 1. Human tissues were stored and analysed in accordance with UK legislation and the Human Tissue Authority licence held by UCL Queen Square Institute of Neurology. Post-mortem samples of brain were sent from Oxford University Brain Bank and Edinburgh University National CJD Research and Surveillance Unit to UCL for analysis.

### Genetic analysis

Blood for DNA extraction from the proband and his sisters was obtained and genomic DNA extracted following appropriate genetic counselling and informed consent. Genomic DNA was later extracted from frozen brain samples from the proband’s father with appropriate informed consent in place from his family. Sanger sequencing of *PRNP* was performed as previously described [36].

### Fluid biomarker analysis

CSF was obtained by lumbar puncture with spinal needle draining directly into polypropylene tubes (no manometer used) and, within one hour of sampling, frozen for RT-QuIC or spun at 2200 g for 10 minutes and then frozen for all other biomarker analysis.14-3-3 protein was detected using immunoblot with enhanced chemiluminescence. S100 Beta (S100B) was measured using an ELISA supplied by Millipore. Total tau, tau phosphorylated at threonine-181 (p-tau181), beta-amyloid 1–42 and 1–40 (Αβ42 and Aβ40) were measured using Lumipulse G kits on Lumipulse chemiluminescence analyser. Νeurofilament light chain (NF-L) was measured using Uman NF-L ELISA for CSF. IQ-CSF RT-QuIC was performed according to previously published methods [29]. Plasma NF-L, glial fibrillary acid protein (GFAP), p-tau181 and p-tau217 were measured using Simoa HD-X analyser.

### Neuropathology and immunoblot

Post-mortem brain tissue was available from the proband’s father. Archival formalin-fixed paraffin-embedded tissue blocks from the neocortex with subcortical white matter, medial temporal lobe region comprising anterior hippocampus, basal ganglia with caudate nucleus and putamen and cerebellar cortex with subcortical white matter at the level of the dentate nucleus were retrieved. The paraffin blocks were reprocessed into paraffin following 1 h pre-treatment in 98% formic acid and stained with haematoxylin and eosin and immunostained for abnormal prion protein (anti-PrP ICSM35, D-Gen Ltd, London, UK 1:1,000; 12F10, Cayman Chemical, UK, 1:200), amyloid-β (DAKO; M0872; 6F3D; 1:50; KG9, TSE Resource centre, Roslin Institute Edinburgh, UK, 1:500), hyperphosphorylated tau (Invitrogen; MN1020; AT8; 1:1200), alpha-synuclein (Abcam; Ab1903; 4D6; 1:1000), anti-phosphoTDP-43 (CosmoBio; TIP-PTD-P01; polyclonal; 1:2000) and p62 (BD Transduction; 610833; 3/P62LCK Ligand; 1:100). Immunostaining was performed on a Ventana Benchmark automated immunohistochemical staining machine (ROCHE Burgess Hill, UK), following the manufacturer’s guidelines, using biotinylated secondary antibodies and a horseradish-peroxidase-conjugated streptavidin complex and diaminobenzidine as a chromogen.

Frozen brain samples (frontal and temporal cortex) were prepared as 10% (w/v) homogenates in Dulbecco’s sterile phosphate buffered saline lacking Ca^2+^ and Mg^2+^ ions (D-PBS, Gibco) using a Precellys Evolution tissue homogenizer (Bertin Instruments). 10% (w/v) brain homogenate (frontal cortex) from a sporadic CJD patient with type 3 PrP^Sc^ (London classification [12]) was used as a control sample. 20 µl aliquots of 10% (w/v) brain homogenate were digested with proteinase K (at a final concentration of 50 µg/mL proteinase K in the sample) for one hour at 37 °C and then processed for SDS-PAGE as described previously [36]*.* Processed samples were analysed on 12% NuPAGE gels (Thermo Fisher Scientific) calibrated using the Seeblue Pre-stained Protein Standard from Invitrogen (Thermo Fisher Scientific) after which gels were electroblotted to Immobilon P membrane (Millipore). High sensitivity immunoblot detection of human PrP was performed using anti-PrP monoclonal antibody 3F4 (Biolegend UK Ltd), which has an epitope spanning residues 104–113 of human PrP, in conjunction with alkaline-phosphatase-conjugated goat anti-mouse IgG secondary antibody (Sigma-Aldrich, A2179) as described previously [36]. All brain tissue was handled according to full UK Department of Health transmissible spongiform encephalopathy precautions [3].

## Results

### Clinical features

The proband is a 50-year-old right-handed white British man who presented with a gradually progressive history of dysarthria, gait disorder and myoclonus. The first symptom was dysarthria, followed around 6 months later by gait abnormality with occasional trips, and another 3 months later by action myoclonus, moderately severe at times, particularly in the lower limbs. The myoclonus responded to treatment with levetiracetam. At the most recent assessment (23 months after first symptoms) he remained relatively mildly affected; he was still going for walks several times per week although he had suffered a few falls and he had given up running. His family described worsening of ‘memory’ for dates and appointments and he stopped working as a project manager around 2 years after symptom onset, after personality change with reduced empathy and patience emerged. His sleep was normal. Sensation, bladder and bowel control and sexual function were normal. He suffers from hypertension and has a one pack year smoking history. He has no other significant past medical history and no risk factors for iatrogenic prion disease.

Positive findings on examination included mild hypomimia and dysarthria with a somewhat monotonous voice. Eye movements were entirely normal. In the limbs, there was increased tone, more marked in the lower limbs with very frequent low amplitude myoclonus superimposed on this. Power was full but movements triggered prominent action myoclonus in upper and, especially, lower limbs. Reflexes were brisk throughout and triggered myoclonus but plantar responses were flexor. Finger–nose test was accurate despite myoclonus but there was some evidence of mild heel-shin ataxia. Testing for dysdiadochokinesia revealed slow but quite accurate movements bilaterally. There was mild bradykinesia with decrement bilaterally. Sensation was normal throughout. Gait exhibited bilaterally reduced arm swing and tandem gait was mildly impaired.

**Fig. 2:**
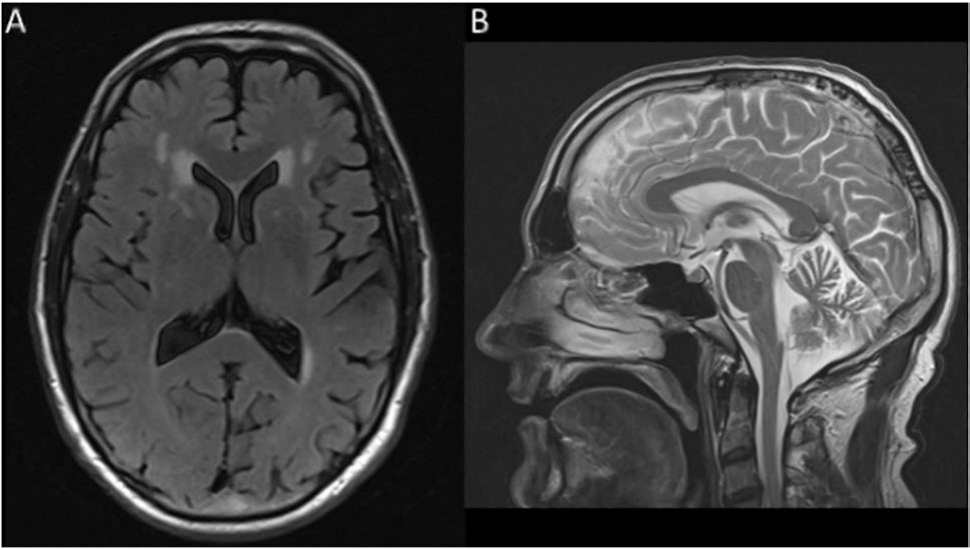
MRI scan of the E146G proband at 9 months following symptom onset. **A** Axial FLAIR imaging demonstrates white matter hyperintensities in excess for age focussed on the frontal lobes. **B** Sagittal T2-weighted imaging demonstrates atrophy of the cerebellum

Bedside cognitive testing with MRC Prion Cognitive Scale [28] revealed reduced verbal fluency (11 words beginning with the letter F in 1 min), and an error on arithmetic (3/4 calculations correct) but naming, memory, digit span, and fragmented letters were normal. Formal neuropsychological assessment revealed moderate executive dysfunction and significantly reduced processing speed, with slightly weak recall memory and confrontational naming. On WAIS-III, he scored 90 for both verbal and performance IQ, which represents severe intellectual underperformance from optimal premorbid estimates. There was mild difficulty copying meaningless, but not purposeful, hand gestures bilaterally. MRI brain (Fig. 2) at 9 months following symptom onset demonstrated significant cerebellar atrophy and white matter T2 hyperintensities in excess of that expected for his age, focussed on the frontal lobes. There was no restricted diffusion. Electroencephalogram (EEG) at 7 months following symptom onset was unremarkable.

The proband’s 46-year-old sister also carries the mutation and has early symptoms: she reports several years of mild impairment of balance in situations such as climbing over rocks but it has not restricted her hobbies which include skiing and horse riding. She has no cognitive complaints. Her cognitive and neurological examination were normal except for subtle gait ataxia detectable only when walking 10 steps in tandem and mild dysarthria. Detailed neuropsychological assessment revealed possible under-functioning relative to optimal pre-morbid estimates. There were slight weaknesses in attentional capacity and processing speed but no other focal deficits. MRI brain and EEG were normal. Another sister, aged 48, carries the mutation but is asymptomatic and has no abnormalities on examination.

The proband’s father died at 55 years of age following an 8-year neurodegenerative illness. This illness began with impairment of balance and coordination, impaired performance at work and personality change with disinhibition, initially manifesting as angry outbursts. Subsequently he developed amnestic symptoms, navigational difficulties and dysarthria. His behaviour became disinhibited and he required institutional care for the final 5 years of his life. Myoclonus was not recalled on family interview as a prominent feature but cannot be excluded as a cause of his impaired ‘coordination’ as he was not examined. Later in the illness he became bedbound, fully dependent and dysphagic requiring a purée diet. Cerebellar atrophy was reported on MRI scan. The diagnosis of prion disease was made on post-mortem examination of the brain and genetic testing was not performed at the time at the request of the family, but has been performed subsequently.

### Genetic analysis

The *PRNP* open reading frame was sequenced and revealed a novel missense mutation of c.A437G (CCDS 13080.1) resulting in substitution of glutamate to glycine at position 146 (p.E146G). This mutation is not reported in GnomAD 4.0.0 or Genebass, and is, therefore, thought to be novel. The codon 129 genotype was valine homozygous in the proband and methionine valine heterozygous in his sisters and his father indicating that the mutation occurred on the codon 129 valine haplotype.

### Cerebrospinal fluid examination and biomarkers of neurodegeneration

CSF testing was performed on the proband and his affected sister and routine CSF constituents including cell count, protein, glucose, and oligoclonal bands were unremarkable. CSF and plasma neurodegenerative disease biomarker profiles are shown in Table 1.

**Table 1:** Fluid biomarker results

	CSF							Plasma			
	S100B (< 740 pg/mL)	Aβ42/40 ratio (> 0.065)	Total tau (146 595 pg/mL)	*P*-tau181 (< 60 pg/mL)^a^	IQ-CSF RT-QuIC	NF-L (< 967 pg/mL)	14-3-3	GFAP (< 142 pg/mL)	*P*-tau181 (< 26.5 pg/mL)	*P*-tau217 (< 0.47 pg/mL)	NF-L (< 21.5 pg/mL)
Proband (first sample)	** > 2000**	0.098	**711**	**84**	Neg	934	Neg	–﻿	–﻿	–﻿	–﻿
Proband (4 months later)	–	–﻿	**–﻿**	**–﻿**	–﻿	–﻿	–﻿	**443**	24.6	**0.56**	8.3
Proband (11 months later)	** > 2000**	0.102	**750**	**88**	–﻿	740	Neg	–﻿	–﻿	–﻿	6.6
Proband (17 months later)	**–﻿**	–﻿	**–﻿**	**–﻿**	–﻿	–﻿	–﻿	–﻿	–﻿	–﻿	6.2
Proband’s sister (first sample)	** > 2280**	0.098	**596**	57	Neg	627	Neg	**685**	22.0	0.18	4.4
Proband’s sister (8 months later)	**1997**	0.109	**600**	54	–﻿	524	Neg	–﻿	–﻿	–﻿	4.5
Proband’s sister (22 months later)	**–﻿**	–﻿	**–﻿**	–﻿	–﻿	–﻿	–﻿	–﻿	–﻿	–﻿	2.5

The reference range for each biomarker is included in brackets. Values in bold are outside the reference range. ^a^ < 58 pg/mL was the reference range for CSF p-tau181 when the proband’s sister’s samples were analysed. *Neg* negative, *NF-L* neurofilament light chain, – not done

### Pathology

**Fig. 3:**
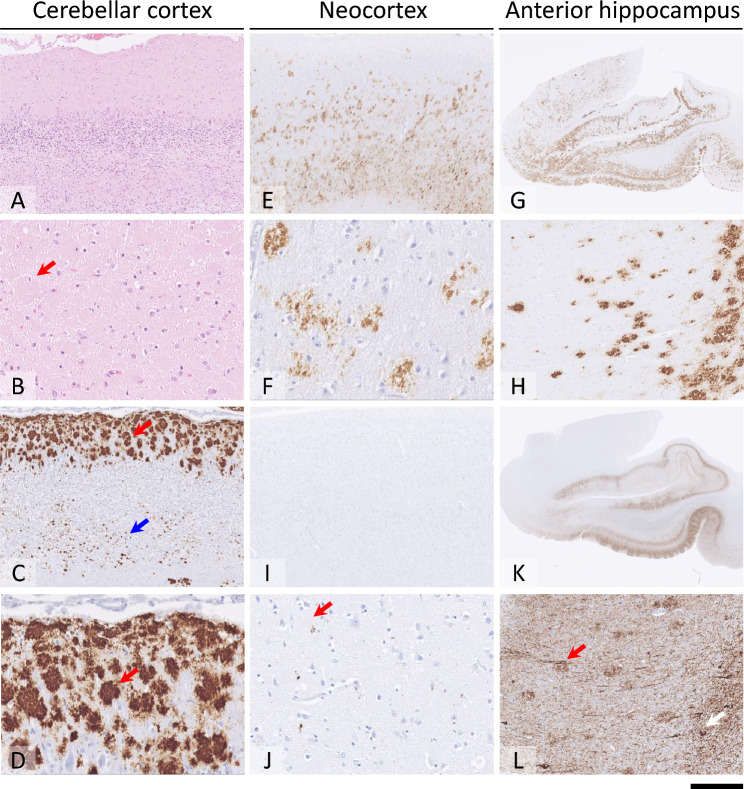
**A** Severe cerebellar cortical atrophy, with marked depletion of Purkinje cells and granule cells is evident on haematoxylin and eosin-stained section. **B** In the cerebellar molecular layer, there are frequent densely packed amyloid plaques (shown with red arrow in **B**). **C** and **D** Immunostaining for abnormal PrP with ICSM35 antibody highlights large plaques in the molecular layer (red arrow in **C** and **D**) and small plaques in the atrophic granule cell layer (blue arrow in **C**). **E** and **F** Less dense PrP amyloid plaques and synaptic abnormal PrP labelling is seen in the neocortex, with accentuation in the deep cortical layers. **G** and **H** shows frequent PrP plaques across the anterior hippocampus and cortex of the parahippocampal gyrus. **I** and **J** In the neocortex hyperphosphorylated tau pathology is restricted to occasional granular deposits in the vicinity of PrP plaques (red arrow in **J**). **K** and **L** In the anterior hippocampus, there is a dense meshwork of tau immunoreactive neuropil threads, PrP associated neuritic plaques (red arrow in **L**) and occasional neurofibrillary tangles (white arrow in **L**). Scale bar: 250 µm in (**A**, **C**, **E** and **I**); 120 µm in (**B**, **D** and **F)**; 300 µm in (**H**, **J** and **L**); 4.5 mm (**G** and **K**)

**Fig. 4:**
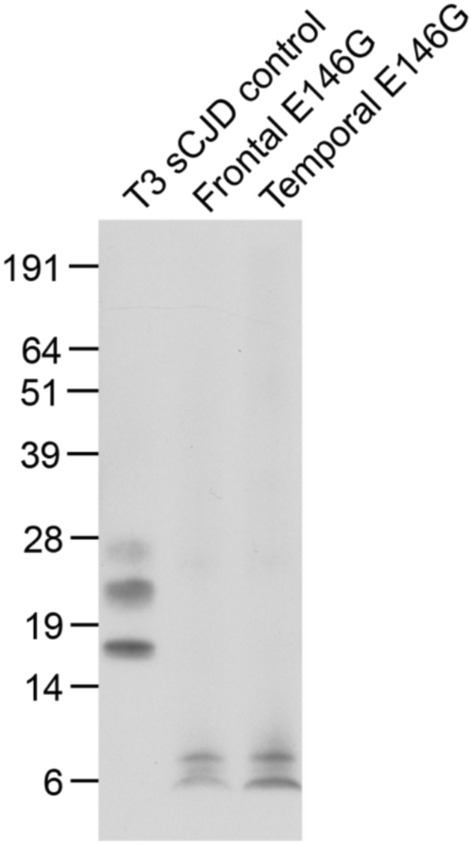
Immunoblot analysis of *PRNP* E146G patient brain for proteinase K-resistant PrP.10% (w/v) frontal and temporal cortex homogenates from the post-mortem E146G patient and 10% (w/v) frontal cortex from a control case of sporadic CJD with type 3 PrP^Sc^ (T3 sCJD control, London classification [12]) were digested with proteinase K (final concentration of 50 µg/mL proteinase K) for 1 h at 37 °C and processed for SDS-PAGE. The immunoblot was analysed with anti-PrP monoclonal antibody 3F4 using high sensitivity chemiluminescence. Loadings for all lanes correspond to 2.5 µl 10% (w/v) brain homogenate. Only a double band of low molecular weight proteinase K-resistant PrP fragments are seen in brain homogenates from the E146G patient

Histological examination showed prominent cerebellar cortical atrophy with chronic gliosis and frequent amyloid plaques seen in the molecular layer, and with widespread Purkinje and granule cell atrophy (Fig. 3A). The dentate nucleus in the cerebellum showed no significant atrophy. Microvacuolar or macro-vacuolar degeneration in the neocortical regions or basal ganglia were not visible, but there were frequent amyloid plaques and chronic gliosis across examined grey matter regions (Fig. 3 B–H). Immunostaining for abnormal PrP confirmed presence of large confluent PrP plaques across the grey matter regions, with particular emphasis in the cerebellar molecular layer (Fig. 3C, D), but sparing of the dentate nucleus. In the deep neocortical layers and in the basal ganglia, there was also synaptic abnormal PrP labelling (Fig. 3E). PrP amyloid angiopathy in the leptomeninges or grey matter was not present. In the subcortical cerebral and cerebellar white matter rare short abnormal PrP threads were evident. Hyperphosphorylated tau pathology was restricted to occasional granular deposits around the PrP amyloid plaques in all grey matter regions (Fig. 3I, J) and to limbic restricted, dense tau pathology comprising frequent PrP amyloid plaque associated neuritic plaques, meshwork of neuropil threads and neurofibrillary tangles, corresponding to Braak and Braak stage II (Fig. 3K, L). Rare Lewy pathology restricted to amygdala was also noted. There was no amyloid-β or TDP-43 pathology. Thickening of the vessel walls of arterioles and small arteries in the cerebral and cerebellar white matter and basal ganglia was noted, with a small proportion exhibiting hyaline change. There was an organising, cavitated infarct in the superior putamen with gliosis and macrophage infiltration in the surrounding neuropil. There was mild diffuse gliosis throughout the white matter examined. Molecular strain typing indicates the presence of a partially protease-resistant PrP fragment double band of low molecular weight (Fig. 4).

## Discussion

The E146G *PRNP* mutation, identified for the first time in this family, was associated with a gradual neurodegenerative disease progressing over around 8 years with variable motor onset (prominent action myoclonus/dysarthria/ataxia) and later cognitive decline. This clinical presentation, particularly prominent lower limb action myoclonus, adds to the phenotypic spectrum of IPD that does not neatly fit into canonical IPD syndromes such as GSS (as originally described in the P102L mutation), familial CJD or FFI. *PRNP* mutations can cause diverse phenotypes, even within the same family, so the extent to which this phenotype is mutation-specific awaits identification of further cases [21, 37]. Symptomatic disease in the proband and his affected sister, and pathological evidence of IPD in the proband’s father, in the context of a novel genetic variant not previously reported in large control population databases is in keeping with inheritance as a highly penetrant autosomal dominant trait [24]. However, the lack of clear family history of the disease prior to the proband’s father is a reminder that not all IPDs present as familial disorders [19, 20], with poor ascertainment of historical cases one possible explanation—the family history of multiple strokes in the sixth decade raises some suspicion here. Accurate diagnosis of slowly progressive IPDs, which may be difficult to identify clinically and are usually RT-QuIC negative, is of increasing importance as rationally designed drugs targeting prion disease enter clinical testing [5, 21, 23], and to facilitate family planning decisions and possible access to pre-implantation genetic diagnosis. This report emphasises the importance of genetic testing including *PRNP* in unexplained neurodegenerative syndromes. Furthermore, it highlights the limitations of NF-L as a test to exclude neurodegeneration in isolation [1], as both symptomatic patients persistently returned normal results for age in plasma and CSF. It also illustrates the potential of an expanded panel of CSF neurodegenerative biomarkers to facilitate nuanced interpretation of pathological processes underlying a clinical syndrome.

Both antemortem patients studied so far exhibit a distinctive biomarker profile. There is persistent elevation of plasma GFAP and marked elevation (> twofold upper limit of normal) of CSF S100B, suggestive of astrogliosis, with no elevation of CSF and plasma NF-L and only mild elevation of CSF total tau. NF-L and total tau are markers of neuroaxonal degeneration, which would generally be expected to be significantly elevated in prion diseases, especially CJD, the most well-studied prion disease, which has a different, more aggressive phenotype [31, 33, 35]. IQ-CSF RT-QuIC is negative, as is most often the case in slowly progressive IPD [25]. The striking CSF biomarker profile of persistently normal NF-L and marked elevation (> twofold upper limit of normal) of S100B has not previously been observed in 62 CSF samples from 50 patients with or at risk of developing IPD in the UK National Prion Monitoring Cohort (unpublished data). We await other reports of E146G IPD with interest to elucidate whether this is a biomarker signature of early E146G IPD. Fluid biomarkers may be used for patient selection and as surrogate endpoints in future clinical trials, particularly in early or pre-symptomatic disease, so characterising mutation-specific differences is crucial for IPDs, a rare but heterogeneous disease group [25].

It is interesting that CSF pTau-181, proposed as a biomarker of Alzheimer’s disease neuropathologic change that is sufficiently specific for diagnosis of Alzheimer’s disease even before symptoms begin in recently revised diagnostic guidelines [17], is persistently elevated (with normal CSF Aβ42/40 ratio) in the proband. Previous data indicate that other IPD with extracellular PrP amyloidosis (F198S and PrP systemic amyloidosis) are associated with tauopathy with neurofibrillary tangles composed of 3R/4R tau with identical filament structures on cryo-EM to those found in Alzheimer’s disease [9, 22], compatible with similar mechanisms of amyloid-induced secondary tauopathy in certain IPD and Alzheimer’s disease. Similar to other IPD with dense PrP amyloid plaques, in the post-mortem case tau pathology with frequent PrP amyloid-associated neuritic plaques, neuropil threads and neurofibrillary tangles were evident, restricted to the medial temporal lobe without any accompanying amyloid-β pathology. Furthermore, hyperphosphorylated tau pathology (usually not neurofibrillary tangles) is a general feature of prion diseases [17], and recent data indicate that in CJD, phosphorylated tau species are also elevated in CSF and plasma as a result of secondary tauopathy [2]. Collectively this highlights a potential pitfall in using phosphorylated tau alone as biomarker evidence of Alzheimer’s disease pathology for diagnosis [17], or for clinical trial enrolment, as in the TRAILBAZER-ALZ 3 donanemab trial [4]. Evaluation of the performance of phosphorylated tau biomarkers in IPD is warranted.

**Fig. 5:**
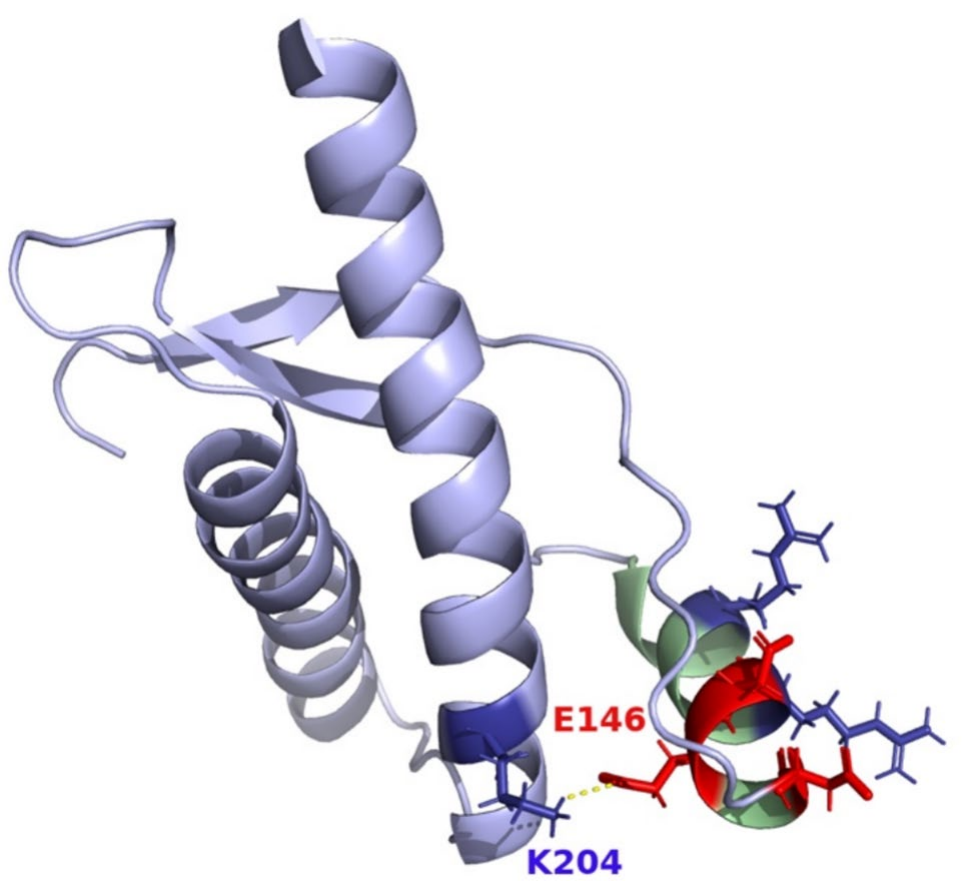
E146G mutation in the context of PrP^C^ structure. Residue 146 is in PrP^C^ helix 1 [15]. Helix 1 is highlighted in green, with the sidechains of negatively charged residues E146 and D144/D147 red, and positively charged residues R148/R151 and K204 blue. The salt bridge between E146 and K204 stabilising the Helix 1 and 3 interaction is shown as a dotted line. This figure was generated using PyMol (Schrödinger, LLC)

Helix 1 of the cellular isoform of PrP (PrP^C^), where E146 resides, has a very high intrinsic helix propensity, in large part due to the pattern of charged residues and associated salt-bridges within it [26, 39]. Salt bridges play key roles in stabilizing the secondary and tertiary structural elements of PrP^C^ in its native conformation [7]. Removal of these charge interactions either through mutation [32] or solution conditions [39] results in the destabilisation of helix 1 and PrP^C^, which facilitates its conversion to a disease-associated conformation [7, 14, 32, 38] The predominance of charged residues within helix 1 also results in an unusually high hydrophilicity [26] and consequent lack of hydrophobic contacts with the other core helices 2 and 3. As shown in Fig. 5, helix 1 has very few long-range interactions, with the salt bridge between E146 and K204 in Helix 3 forming a key part of a highly conserved electrostatic network that stabilises the association of helix 1 with helices 2 and 3 [15]. E146G mutation is likely to result in the destabilisation of Helix 1 through the loss of negative charge and favourable electrostatic interactions within the helix 1 dipole. In addition, the loss of the salt bridge with K204 will significantly reduce the degree of stabilising interactions between helix 1 and helices 2 and 3. The substitution of glycine in particular, an amino acid with effectively no side-chain, would be predicted to increase the conformational flexibility and entropy within this region of the protein, potentially facilitating conformational conversion. All of these factors suggest that this mutation will destabilise PrP^C^ and assist its conversion to the disease-associated form.

Immunoblot analysis of proteinase K-resistant PrP from post-mortem patient cortex demonstrates only a proteinase K-resistant PrP doublet of low molecular weight, and no PrP^Sc^ fragments at 21–30 kDa, as seen in sporadic, variant and iatrogenic CJD. Similar low molecular weight fragments have been reported in variably protease-sensitive prionopathy (VPSPr) and IPD, mostly point mutations, including F198S, P102L, A117V, D178N, and 7-OPRI but not E200K which has a sporadic CJD-like phenotype [11, 18, 30, 34]. In P102L IPD the abnormal PrP conformer generating the low molecular weight fragment has previously been detected in areas of brain with PrP amyloid plaque formation [30], in keeping with the extensive PrP amyloidosis seen in this case. In contrast PrP^Sc^ generating 21–30 kDa fragments (similar to that seen in sporadic CJD) are detected in areas of P102L IPD brain with a synaptic pattern of PrP deposition and spongiosis [30], and this histological pattern was not prominent in this case. Recent cryo-electron microscopy structural characterisation of PrP amyloid from F198S IPD brain indicates that the core of the PrP amyloid fibril comprises residues 80–141, although mass spectrometry and immunochemistry data indicate that amyloid deposits consist of a diverse population of peptides with a ragged N terminus around residue 80 and ragged C terminus between residues 149–170, and species extending to N and C terminals of full length PrP have also been described [10]. The double band of low molecular weight proteinase K-resistant PrP fragments seen on immunoblot of E146G IPD brain would be in keeping with a protease-resistant amyloid core with ragged termini and suggests that the E146G mutation leads to the formation of a disease-associated PrP amyloid with a core encompassing similar PrP residues to the PrP amyloid seen in F198S IPD.

## Conclusion

We report the first pedigree of IPD due to *PRNP* E146G mutation, with inheritance as an autosomal dominant trait with high penetrance. The clinical phenotype is a gradually progressive neurodegenerative disease, which exhibits variable motor onset (prominent lower limb action myoclonus/dysarthria/ataxia) and later cognitive decline. Post-mortem confirms IPD with profuse large PrP amyloid plaques across cerebral and cerebellar grey matter and low molecular weight protease-resistant PrP fragments on immunoblot. We highlight a distinctive fluid biomarker profile in cases so far, including persistently normal CSF and plasma NF-L with very elevated CSF S100B and plasma GFAP, and discuss the broader clinical relevance for biomarker interpretation.

## Data Availability

Source data can be made available upon reasonable request but may require a data transfer agreement.
